# Supplementary material for: Are carbon emissions trading and green financial instruments synergistic? -Comprehensive quantitative research based on content analysis
Source: PLoS One. 2024 Mar 7;19(3):e0298601. doi: 10.1371/journal.pone.0298601 (PMC10919601; doi:10.1371/journal.pone.0298601)
Supplement: S1 Data — (ZIP) [file pone.0298601.s001.zip › Data/地级市Z_F工作报告.zip!/╣π╢1⁄2/╟σ╘╢╩╨/└┤╫╘╣1⁄2╓┌║┼í╛┬φ┐╦╩2╛▌═°í┐_2014.docx]

各位代表：

我代表市人民政府，向大会报告政府工作，请予审议。并请政协各位委员和其他列席人员提出意见。

2013年工作回顾

刚刚过去的2013年，是清远转型发展取得实质成效的一年。在市委的领导下，在市人大、市政协的监督支持下，我们坚持转型发展与加快发展并举，切实转变发展方式，努力扭转经济下行态势，实现了经济增长的触底回升和发展质量的提升，完成了市六届人大三次会议确定的年度工作目标。全市实现生产总值1093亿元，同比增长8.2%；人均生产总值2.89万元，增长7.4%。来源于清远的财政总收入246.6亿元，增长18.82%；全市公共财政预算收入92.8亿元，增长6.8%；其中市本级公共财政预算收入27.87亿元，增长7.4%。固定资产投资增长15.5%。社会消费品零售总额增长10.7%。城乡居民人均收入分别增长9.5%和12.2%。在经济环境错综复杂，又连续遭遇两场特大洪涝灾害的情况下，能实现上述目标，实属不易。

刚刚过去的2013年，也是市政府工作在重点领域取得突破性进展的一年。我们紧紧抓住交通、城市、产业和民生四项关键工作，努力突破制约清远发展的瓶颈，重点工作取得了重大进展：广清城际轨道开工建设；广清高速扩建清远段率先建成；一批损毁严重的国省道完成重建改造。燕湖新城主干路网的征地拆迁基本完成，首批工程全面铺开；江滨公园等城市提质工程顺利建成；成功创建成为“国家园林城市”。产业园区的配套取得明显突破；一批投资大、带动力强的产业项目落户清远。中心城区和偏远地区的学校布局调整基本完成；全市高考重本和大专上线率的增幅均跃居全省首位。广清合作发展的机制已经建立，广清一体化发展的格局初步形成。

过去的一年，我们主要抓了以下六项工作：

一、扎实推动经济转型提速

通过完善机制加快重点项目建设。成立了市重点项目建设办公室及用地、征地拆迁、环评、融资、招投标和政府采购等5个专责小组，明确领导责任和时间节点，并通过定期跟踪督办、优化再造流程、强化用地和资金保障，多策并举推动重点项目建设。省、市重点项目共完成投资290.3亿元，占年度投资计划的96.2%，其中省重点项目完成年度投资计划的108.2%。

瞄准带动力强的项目招商引资。强化招商工作统筹，引进了一批带动力强的企业。全市共引进产业项目420个，增长65.4%；计划投资1549.5亿元，增长203.2%，累计实际投入162.6亿元，增长54.6%。其中，长隆集团计划投资300亿元打造世界级的森林旅游综合体；天安集团计划投资50亿元打造区域性科技产业综合体；金发科技公司计划投资38亿元打造高性能改性塑料生产基地。

下大力气帮扶优质企业。召开60多次政企座谈会，实施“百家企业帮扶工程”，采取“一企一策”，由市、县领导挂点服务重点企业，研究解决企业发展遇到的问题。落实各项税收优惠政策，共为企业减免税负5.6亿元。开通中小微企业“助保贷”﹝1﹞业务，金融机构支持中小企业贷款金额387.4亿元，4家企业在股权交易中心注册、挂牌，东鹏陶瓷控股在香港联交所上市。

积极推动产业转型升级。完成11条水泥生产线的脱硝治理和7家陶瓷企业的综合整治，淘汰一批落后产能。新增高新技术企业12家，新认定省级创新型企业3家。国家级工程中心实现零的突破。42所高校、科研院所的128名科技特派员进驻74家企业。新增9家银行、证券和保险分支机构，清新惠民村镇银行申请开业，世界500强永旺公司后台信息处理项目正式运营，华康私募股权基金项目落户。三次产业增加值的比重调整为15：40：45。

二、努力提升发展的基础条件

推进交通体系建设。全市交通建设投资86亿元，完成年度计划的119.4%，其中高速公路投资71.8亿元，完成年度计划的111.3%。广清高速公路扩建清远段建成通车，广乐、二广高速公路超额完成年度建设任务。佛清从、连佛、广清与清连高速连接线和汕湛、汕昆高速公路清远段的前期工作进展顺利。广清城际轨道先行工程全面施工。完成了清三、清佛公路、106国道佛冈县城以南段等国省道改造工程。北江千吨航道工程前期工作基本完成，清远港港澳航运顺利复通。

加快园区配套建设。重点园区扩园取得突破性进展，五大产业园﹝2﹞累计投入开发资金46.89亿元，已开发面积约5.2万亩，完成了一批主干道路，以及供水、供电、供气、污水管网设施的建设。广州（清远）产业园前期工作启动。

改造升级城乡电网。完成了清城、清新、佛冈和“两德”经济合作区电力专项规划与城市规划的衔接，推进“一镇一册”﹝3﹞配电网规划修编。共完成电网固定资产投资12.61亿元，建成投产35千伏及以上输电线路206.8千米，新增变电容量150万千伏安。500千伏贤令山主变扩建、220千伏石潭变电站等一批输变电工程完成并运营。投入2.7亿元完成了635个农网改造升级项目，直接惠及6万农户、约30万人口。

巩固良好生态基础。整合资金5亿多元，推进生态景观林带和森林碳汇﹝4﹞林建设，完成造林作业面积约31.4万亩、森林抚育66.5万亩，新增生态公益林31万亩，生态林面积占林业用地的40.4%。查处非法采矿案件112宗，关闭采石采矿场26个。治理规模化禽畜养殖场27家。初步建成横荷、乐排河污水处理厂，完成90多公里的污水管网建设。北江水质总体稳定。整治涉重金属企业和项目56个，对5家违规企业实施关停。抓好机动车尾气污染治理，开展PM2.5﹝5﹞监测，市区空气质量总体良好。

启动智慧城市﹝6﹞建设。出台智慧清远建设行动计划，积极推进宽带普及提速，城市及农村带宽接入能力分别达到20兆和4兆以上。行政村实现3G网络、光纤宽带全覆盖，住宅小区光纤覆盖90%以上，公益性机构光纤到达率82%。有线数字电视网络双向化改造完成98%。4G网络﹝7﹞建设正式启动。建立了全市统一的电子政务平台，实现了清远市民卡公交应用系统与“岭南通”﹝8﹞的全面对接。积极推广应用数字清远地理信息公共服务平台。

三、全面铺开“一心两核”﹝9﹞城市建设

加快推进新区各项基础工作。完成了燕湖新城45平方公里的总体规划，基本完成起步区控制性详细规划和核心区详细设计，实现了城市规划与土地利用规划的统一。燕湖新城第一、第二批市政道路涉及的征地拆迁基本完成，34个总投资237.57亿元的项目全部完成前期工作，其中开工建设7项，交付使用2项。英德东岸新城4.59平方公里的起步区已开工建设，2条主干道正在抓紧施工，5条主干道前期工作进展顺利。连州新区完成三江河以南控制性详细规划的编制，兴连大道主体工程已经完成。

提升城市环境功能。实施城市提升“六大工程”﹝10﹞，对市区各类绿地进行改造提升，完成了飞来湖公园、江滨公园等一批园林景观工程改造。大力推进第二水厂建设和七星岗水厂取水口迁移工程，清城、清新供水实现一体化。完成了13个肉菜市场的升级改造，将4.6万盏路灯更换为LED﹝11﹞节能灯。规范市区公共汽车标志标识设置，优化候车亭规划。大力推进生活垃圾无害化处理设施建设，全面开展内街小巷环境卫生专项整治。严厉打击车辆超限超载违法行为。抓好广清高速公路出口、广清大道沿线、白庙渔村、城西大道迳口段等重要节点的综合整治。顺利通过省卫生城市复检。

完善城市管理体系。成立城市管理委员会，构建“大城管”格局。将市区交警大队下放清城区管理，将环卫清扫保洁、道路绿化养护、公园广场管理、市政设施维修等城市管理事务推向市场。完成了数字城管系统平台的安装并在市区范围内开通试运行。

四、从改革入手推动农村发展

推动农村综合改革。14554条自然村成立了村民理事会。在阳山县6个村试点推进土地承包经营权确权登记颁证，英德市农村集体土地流转服务中心、清新区三坑镇农村土地承包经营权流转中心试点工作进展顺利。启动了农村金融改革创新综合试点工作。阳山县被列为全国农村综合改革示范试点，佛冈县成立了全国首家在经济联合社框架内设立的农民信用合作组织。

启动美丽乡村建设。完成101条美丽乡村的建设规划工作，初步建立美丽乡村建设工程项目库。建成“一镇一站”垃圾中转站84个、“一村一点”垃圾收集点15790个，农村改厕普及率达82.2%。完成4条村的整村推进建设，新创建省级卫生村9条。

实施农业“三品”工程。引进50多个农业新品种进行示范试验。茶业、草食动物、北江特色渔业、花卉苗木等新四大支柱产业发展迅速。新增“三品一标”﹝12﹞农产品、农民专业合作社、省重点农业龙头企业一批，中国驰名商标2件。大北农科技集团广东科技园落户我市。13家企业被评为广东省“菜篮子”基地。

推进农田水利建设。投入5.33亿元推进高标准农田建设，完成了44.42万亩建设任务和37.86万亩的前期工作。362宗民生水利工程已开工335宗、完成231宗。2201宗水毁水利工程完工1589宗。新增农村饮水安全工程全面完成。市区清东围大型泵站改造工程已完成2个，在建1个，大燕河整治主体工程和市区电排站水闸改造工程基本完成。

实施新一轮“双到”扶贫。444名干部进驻重点帮扶村，基本完成284个重点帮扶村3.1万贫困户的建档立卡。积极做好国家级扶贫改革试验区建设前期工作。完成“两不具备”贫困村庄搬迁安置任务，实现“五年任务三年完成”目标。1607户因灾全倒户全部搬入新居。加快解决石灰岩地区外迁人口安置遗留问题。开展连南扶贫小额贷款试点工作。

五、以教育医疗为重点推进基本公共服务均等化

调整布局推动教育均衡发展。投入11.4亿元，完成市中心区域12所学校的新建和改扩建，新增优质学位1.84万个。完成村小改造48所、教学点改造96个，恢复偏远地区教学点11个和完全小学4所，全市农村和边远山区义务教育阶段学校教师岗位津贴人均每月提高500元。义务教育标准化公办学校覆盖率达97.2%，同比提高15.7%。免除中职学校农村学生、城市涉农专业学生和家庭经济困难学生学费，解决进城务工人员随迁子女中考升学问题。广外附属学校、华师附中进驻我市设立分校。省级职教基地规划通过评审，岭南职院、碧桂园职院开工建设。成立了市教育教学研究院，推行校长聘任制。创建成7个省教育强县（市、区）、63个省教育强镇（街、乡），教育强市创建工作通过省初评。学前教育毛入学率达93%，高考取得历年最好成绩，全市在校中小学生实现了从净流出转向净流入﹝13﹞。

大力改善基层医疗卫生条件。完成256条村卫生站标准化建设和三批基层卫生骨干的培训任务，解决基层医疗服务机构债务和在岗不在编人员待遇问题，启动了镇村医疗机构一体化管理工作。创建6家平价医院和21家平价诊室，99家基层卫生院和1000多个村级卫生站提供平价药包服务。清城区洲心社区卫生服务中心创建为“国家示范社区卫生服务中心”。公立医院试点改革和市级公立医院“去行政化”改革进展顺利。率先开展市县医疗机构一体化管理探索，市中医院全面托管阳山中医院，市人民医院全面托管佛冈医院集团﹝14﹞。

抓好以十件实事为重点的民生改善。各级财政投入11.3亿元，全面完成了十件民生实事﹝15﹞，解决了一批事关群众利益的热点难点问题。城镇新增就业、失业人员再就业、农村劳动力转移就业均超额完成年度任务。高校毕业生登记就业率98.3%，城镇登记失业率下降到2.3%。提前超额完成了城镇职工基本养老、医疗、失业、生育、工伤和城乡居民养老保险扩面征缴任务。对24.46万人次实施了医疗救助。共建成各类保障房7064套，完成率达124.9%。全年民生领域支出130.5亿元，基本公共服务支出78.6亿元，分别占公共财政预算支出的70.3%、42.4%。

六、努力改善营商环境

通过改革提升政府效能。在粤东西北率先启动商事登记制度改革，全市新登记各类市场主体2.27万户，增长15.8%。继续推进行政审批制度改革，市级政府取消行政审批事项102项、转移47项、下放60项、委托管理96项。推进网上办事大厅市县一体化建设，全市6211项办理事项进驻市县两级网上办事大厅，逾六成业务实行网上办理，办事效能总体提速54.6%。推进12345政府服务热线平台建设，整合非应急类服务热线27条。

以“两建”﹝16﹞完善市场经济秩序。在公务员、律师、医务人员等群体和建设工程、税务等领域开展社会信用体系建设。推广再生金属行业信用体系建设经验，推进农户信用信息收集和信贷应用。从旅游市场、出租汽车经营等15个重点领域入手，整治潜规则，优化企业经营环境。建成药品电子监管体系，基本药物生产和批发企业、基本药物生产配送品种全部实现电子监管。建立了农产品质量安全监管机制，农资经营点全面落实进销货台账制度和索证索票制度。8个市场开展了创建诚信市场活动。重视发挥政府法律顾问的作用，共有102人次参与处理各类法律事务98宗，并跟踪服务90项市重点项目建设。

加快社会组织培育发展。落实扶持政策，降低准入门槛，发挥枢纽型组织作用，建设孵化基地，新增社会组织164家。将涉及11个行政单位共47项转移事项交由社会组织承接，涉及政府部门向企业提供服务的30项事项委托给相关协会和商会。

过去一年，我们大力加强政府自身建设，严格执行中央“八项规定”﹝17﹞，向社会公开“三公”经费﹝18﹞。深化非税执收单位经费供给改革，实现了非税收支完全脱钩管理；成立公共资源交易中心；加强对重大项目和重点资金的跟踪审计，强化经济责任审计监督；清理了市级考核检查评比表彰项目；强化了廉政风险防控。完善新闻发言人制度，拓宽政务公开渠道，主动接受人大、政协的监督，鼓励和支持社会公众、新闻媒体对政府工作进行监督。

我们还全力抗击了“5·15”、“8·16”特大洪涝灾害，有效防控了H7N9流感疫情。扎实推进“平安清远”创建工作，进一步加强了社会治安综合治理，加大了安全隐患排查和规范化建设力度，确保了社会大局稳定。成功承办了2013广东国际旅游文化节，并以此为契机加强城市营销，有效提升了清远的城市形象。成功举办了第五届全市运动会。完成广播电视“户户通”工程任务。全面贯彻民族宗教政策，民族、宗教关系保持和谐。国防建设和民兵预备役工作不断加强，军民共建、双拥、优抚安置深入推进，军政军民更加团结。

各位代表，过去一年各项成绩的取得，是在市委领导下，全市上下同心协力、攻坚克难的结果，也是方方面面鼎力支持的结果。在此，我代表市人民政府，向所有关心、支持、参与清远建设和发展的各界人士和朋友们，致以衷心的感谢和崇高的敬意！我特别要感谢驻清部队和武警、消防官兵，感谢公安、电力、电讯、供水和交通公路系统的干部职工，在去年两次特大洪灾中，他们第一时间赶赴灾区一线，确保了人民群众的生命财产安全，及时恢复了灾区正常的生产生活秩序，为地方经济发展和社会稳定提供了坚强有力的保障。

在梳理成绩的同时，我们也冷静分析了工作中存在的问题，主要有五个方面：一是，产业升级还没有取得根本性突破，产业园区配套水平低，新引进的优质产业项目少，转型升级和加快发展都面临较大压力。二是，主体功能区建设还没有取得实质性进展。北部地区如何实现绿色发展，中南部地区如何更好地融入珠三角，还缺乏更有效的措施和办法。三是，营商环境仍然存在不少问题，潜规则在一些领域依然存在，行政效率还不适应企业发展，与珠三角的交通对接薄弱，贫穷偏远的城市形象还没有从根本上得到改变。四是，生态环境保护压力加大，部分地区的环境容量已经接近极限，个别地方已经超越了生态底线，要在加快发展的同时保住青山绿水，面临很大压力。五是，城乡、区域发展不平衡问题依然突出，偏远地区的基本民生保障水平仍然很低，全市还有18万相对贫困人口。对于上述问题，我们将在今年和今后的工作中，尽最大的努力务实解决。

2014年工作安排

各位代表，本届政府就任于清远经济社会发展关键的转折期，我们承担着三项基本的使命：第一，从交通、城市和产业园区入手，根本上改善清远的发展条件，推动清远经济社会进入新的发展阶段；第二，从提升基本公共服务均等化入手，促进区域协调发展，为全面建成小康社会创造条件；第三，从转变经济发展方式入手，在提升经济发展质量的同时，巩固和发展清远的生态优势。通过前两年的努力，这三项任务都取得了积极的进展。我们完全有信心再用三年时间，基本完成上述三项任务，实现任内五年的预期目标。可以预见，到2016年底，我们可以基本建成连接珠三角的城际轨道和多条高速公路；基本完成燕湖新城和英德、连州新城区的框架建设；基本完成市县两级主要产业园区的配套建设；基本实现民生保障均等化的首期目标。到那个时候，清远的发展条件将发生根本性变化，全市上下对清远的发展前景也会更加自信自豪，清远将步入更加健康、协调、可持续的发展轨道。因此，做好本届政府后三年的工作至关重要。今年是承上启下的一年，也是后三年工作的关键一年，必须深入贯彻落实十八届三中全会、省委十一届三次全会和省十二届人大二次会议精神，按照市委六届六次全会的总体部署和政府工作的具体安排，认真落实好今年的各项工作。

综合研判，尽管今年面临的挑战依然很多，但经济大环境总体好于去年：十八届三中全会全面深化改革的部署，将进一步激发发展的活力；省委、省政府大力促进粤东西北地区振兴发展，将极大改善清远的发展条件；广州的全面对口帮扶，将有力助推我们加快发展。特别是，经过近几年的务实调整，全市上下科学发展的思路更加清晰，振兴发展的愿望更加强烈。只要我们坚持稳中求进，注重改革创新，坚守绿色发展，善于把握发展中的机遇，注意规避发展中的风险，完全有可能在新的一年，实现更好更快的发展。

因此，今年的政府工作应该把握好四个关键点：第一，加大固定资产投资和产业发展力度，推动经济实现有质量的较快增长；第二，扎实推进重点领域改革，始终以改革的思维解决发展中的问题；第三，借势广清一体化发展，通过服务广州和接受广州的辐射加快发展；第四，把基本民生保障摆在更加突出的位置，在市域基本公共服务均等化上下功夫。

基于以上研判和考虑，建议今年经济社会发展的预期目标和约束指标是：生产总值增长10%；固定资产投资增长18%；地方公共财政预算收入增长10%；社会消费品零售总额增长13%；城乡居民人均收入增长10%，城镇居民登记失业率控制在3.5%以内，居民消费价格总水平上涨幅度控制在3%以内；城镇化率提高到49%；社会保障、资源环境保护、节能减排及安全生产完成省定目标。

上述预期目标的建议，是经过认真分析和测算后提出的，既有实现的可能，也需要付出艰辛的努力。需要说明的是，今年经济增长目标的建议，比前两年更加积极，主要是基于两个方面的考虑：一是，经过近几年的调整，我市经济增长已经触底回升，加上一批新的产业项目将形成产能，今年有可能实现较快的增长。二是，发展不足，经济总量过小，仍然是清远当前的主要矛盾，我们要在2020年与全国同步建成小康社会，也必须努力加快发展步伐。

围绕实现上述目标，建议今年重点安排八项工作：

一、巩固和发展经济企稳回升的良好态势

以更大力度推进重点项目建设。安排市重点项目238项，总投资1968亿元，年度计划投资362亿元；列入省重点建设项目31项，总投资785亿元，年度计划投资147.4亿元。进一步压减重点项目审批时限，完善市县两级班子成员联系督导、部门联席会议和建设管理考评机制，加快推进108项续建项目，确保52项在建项目建成投产。力争78项新安排项目下半年全部进入实质性施工。同时，做好下年度重点项目的前期工作。

加大优质企业帮扶力度。落实“推进百家企业帮扶工程”和“一企一策”帮扶措施，建立制度化的政企联席会议机制，健全投诉处理和重点企业跟踪制度，及时帮助企业解决实际困难。完善“政银企担”合作机制，继续引进和设立创业投资基金，协助开展中小企业集合债、私募债等金融创新业务。继续推动企业改制上市，支持上市后备企业做大做强，鼓励符合条件的企业到“新三板”上市。推进信息化和工业化融合。抓好人才政策的落实，制订出台技能型人才引进优惠政策。探索科技金融的融合发展，推动技术专利质押贷款工作。

推进产业园区扩能增效。加快五大产业园项目用地清理和整合工作，力争上半年完成广州（清远）产业转移园扩园申报、民族工业园创建省产业转移园工作，年内完成佛山顺德（英德）产业转移园、佛山禅城（清新）产业转移园扩园申报工作。加快石角制造业基地扩容和申报省级产业园工作。继续推进园区道路、电力、通讯、排污等基础设施和生活类服务设施建设。加强园区社会事务管理和相关公共服务。通过配套商业用地开发、委托基础设施项目建设、土地整理等方式，引导更多社会资金参与园区开发建设。计划四个省级产业园区引进项目86个，完成固定资产投资73亿元，完成设备投资22亿元，实现规上工业增加值85亿元，税收11亿元。

开展更有针对性的招商引资。制订务实有效的招商引资计划，完善招商引资信息和利益共享机制，开通清远招商网，开展小分队驻点招商和重点企业拜访活动，组织开展各项经贸合作活动，重点抓好珠三角特别是广州的招商推介活动。充分依托现有制造业龙头企业，引进上下游企业。通过“补链、强链”，推动陶瓷、再生金属等传统产业转型升级。大力引进呼叫、物流、旅游和金融后台等现代服务业。加强已引进和洽谈项目的跟踪工作，力争40个亿元以上筹建项目开工建设、50个亿元以上洽谈项目签订合约。帮助企业解决好用地、用电等问题，力促现有企业增资扩产。

二、扎实推进重点领域改革

深化行政审批制度改革。完成市、县两级政府机构改革，建立政府部门权责清单。继续下放、转移、委托和取消行政审批事项，一般性经营项目不再进行审批并实行自动备案，一般性技术审查事项全部推向市场，外资企业年度联合年检实行报告制。将建设工程项目涉及的审批事项归入“五个集装箱”﹝19﹞，分别由牵头部门组织实施并联审批。上半年完成部门审批事项向单个科（股）室集中，审批科（股）室向行政服务中心集中。在全市范围内推行商事登记制度改革，完成负面清单﹝20﹞和监管清单的制订，建设商事主体登记管理信息平台，形成有效的后续监管机制。将网上办事大厅延伸至镇（街），并整合成集商事登记、政务服务、公共资产交易等功能为一体的信息共享平台。推行科技创新券﹝21﹞。

深化农村综合改革。稳步推进村建试点改革，完善村级民主自治机制。全面完成村委会换届选举，加强基层组织建设。总结推广“叶屋经验”和“新城经验”，解决耕地经营碎片化问题。开展土地承包经营权确权登记颁证，建立农村土地经营权流转、农村集体产权管理服务交易平台。完善农村金融信用体系，推广清新区“政银保”信贷模式，鼓励各地试点探索建立农村集体经济联合社信用合作部。大力发展政策性涉农保险，鼓励保险机构开发家禽、生猪养殖及蔬菜、水果、茶叶种植等规模产业保险项目。

深化社会民生领域改革。创新民生项目规划、决策和投入机制，试行市民“点菜”、政府“配餐”模式，满足多样化的民生需求。抓好枢纽型社会组织孵化基地建设，支持和发展社会志愿服务组织，加快行业协会去行政化。完善校长聘任制改革，全面启动督学责任区工作。完善清远日报社运行机制改革，稳妥推进清远广播电视台体制机制改革。推进机关事业单位养老保险制度改革。实行公务员职务与职级并行，职级与待遇挂钩。加快推进劳动保障监察行政综合执法改革，开展构建和谐劳动关系综合实验区试点。推进事业单位分类改革。加快公立医院改革，发展民营医院，推行合理分级诊疗。完善基本药物制度和药品交易制度。实施“病种分值”结算办法。探索城市居家养老与社区服务相结合的模式，开展农村居家养老“幸福计划”建设试点，推行“公建民营、民办公助”的养老服务机构运营模式。建立统一的城乡居民基本养老保险制度。全面放开县城和中心镇落户限制，有序放开中心城区落户限制。

三、全力组织交通建设大会战

着力完善快速交通体系。把高速公路建设，特别是与珠三角高速公路体系的充分对接，作为今年基础设施建设的首要工作。确保广乐、二广高速公路年内建成通车，实现“县县通高速”目标。力争佛清从高速公路进入实质性施工，基本完成广清与清连高速连接线和汕湛高速公路前期工作，启动汕昆高速公路征地拆迁工作并确保先行工程动工，完成连佛高速公路项目核准和初步设计审批。全面完成广清城际轨道征地拆迁工作，上半年全线铺开一期工程建设；积极推进二期工程申报立项，争取早日动工。

全面铺开国省道改造。启动和推进国道106线佛冈北和英德段，国道323线连南、连山、阳山段改扩建，省道259线连州段、省道354线佛冈段等第二批25个、600公里的国省道路面大修和升级改造工程。继续加快连怡一级、英坑二级和清四一级公路等国省道的路面大修，确保年内完工。启动一批县道路面维修和改造项目。将清佛一级公路洲心收费站迁移纳入广乐高速次票管理。力争北江五桥完成桩基建设，北江四桥正式动工，清城石角至清新山塘跨江大桥及南北连接线完成前期工作。继续开展以源头治理为重点的“铁腕治超”行动。

建设北江千吨航道。加快推进北江千吨级航道升级改造工程，深入推进北江绿色水运示范项目建设，开展飞来峡、清远水利枢纽二级船闸项目前期工作。加快清远港迁建前期工作。抓好北江两岸老旧码头改造，规范简易码头和临时作业点。

四、务实推进城市的扩容和提质

全面铺开燕湖新城建设。加快推进人民东路、北江东路、迎宾大道、燕湖大道等26条骨干道路和5个公寓式安置区的建设，启动起步区25条支路的建设。加快110千伏洲心变电站等输变电工程建设，全力推进燕湖大道高压电力线路迁改工作。全面铺开燕湖新城水系建设，加快大燕河、青榄海整治和大燕湖工程建设。启动广清城际轨道清远站TOD﹝22﹞城市综合体建设。通过引进呼叫和金融保险后台等产业、推进户籍制度改革、吸引外出务工回流群体落户等措施，扩大中心城区人口规模。启动低碳生态示范城区建设。

加快英德、连州新城建设。推进英德东岸新城起步区的开发建设，新开工5条城市主干道和小学、排涝泵站、湿地公园、安置小区的建设，力争起步区主干路网基本成型。完成连州新区城西控制性详细规划编制，加快迎宾大道等一批市政道路建设，铺开城南体育公园、沿河绿化景观带、文化公共配套场馆等设施的建设。力争用三年时间，完成英德、连州新城的框架建设。

提升现有城区功能环境。继续抓好中心城区“五城同创”﹝23﹞工作。从调整学校布局、增设停车场、改造旧街区、增加休闲运动场所等方面入手，推进现有城区的环境功能提升。完成连江路等5条道路及一批交叉口整治，规划建设餐饮一条街。开工建设市区第二水厂。启动海仔排涝渠首期整治工程。完成全市227公里，其中中心城区40公里的绿道建设。加快智慧城市建设，整合“智能交通”、“数字城管”、“平安清远”资源，构建中心城市综合监管平台。完善公交首末站、停靠站建设，加快推进市区生活垃圾焚烧厂和青山生活垃圾无害化填埋场二期工程，推行垃圾分类。抓好内街小巷、建筑工地环境专项整治，推进重要节点绿化景观改造，规范门店招牌管理。加快推进市区建筑材料、陶瓷原废料密闭运输。

推进县城和中心镇提质。完成县城和中心镇的规划修编工作。利用土地规划中期评估机会，做好土规调整，实现“城规”对“土规”的全覆盖。实施县城和中心镇绿化、美化、亮化、净化工程。加快推进各县（市、区）垃圾处理设施建设。严格城镇规划管理，重拳整治违法建设行为。全面放开县城和中心镇落户限制。向县城和中心镇集中优质教育、医疗资源，引导农村人口向城镇有序转移。

五、大力改善农村生产生活环境

因地制宜建设美丽乡村。扎实开展县（镇）域村庄现状调查，做好村庄整治规划。大力清理农村违法建设用地，清拆危旧泥砖房。完成首批253个村庄的规划、76个村庄的提升建设。完成广清高速、清连高速沿线村庄建设规划，推动环境风貌整治。进一步推进农网改造升级和乡村绿化。支持和指导佛冈新农村试验区建设。

完成一批民生水利工程建设。启动连江流域防洪体系建设规划和设计工作，开展大燕河沿线堤防升级工程前期工作，上半年完成清东围大泵站改造工程。铺开新一轮高标准基本农田建设，加快清新、英德省级水利建设示范县和连州、阳山等7个农田水利建设重点县建设进度，确保工程质量。确保6宗小型病险水库除险加固工程、18宗机电排灌工程汛期前完成建设任务。完成50 宗小型灌区续建配套与节水改造工程建设，力争启动村村通自来水工程前期工作。

加大“三品”工程推进力度。完成5万亩沙糖桔病残园的清理工作，抓好沙糖桔替代品种的政策扶持。完成农产品二维码信息体系建设。新制定清远桂花鱼、清远山羊等一批地方标准。在巩固传统优势品种、强化原有农业品牌的基础上，将茶业、桂花鱼、山羊、清远鸡品牌打造成为清远农业的“四张名片”。 推动供穗蔬菜“净菜上桌”和精加工，增加农产品附加值。选取3个大型农机专业合作社（联社）开展农机社会化服务试点工作。大力推广农产品电子商务交易。重点推进大北农科技集团广东科技园、连南稻田养鱼示范点等项目建设。加强全省现代农业示范区（佛冈）建设。制作“三品”工程、农业龙头企业专题宣传片，出版茶叶文化主题书籍。推进农业科技园﹝24﹞建设。

抓好扶贫改革试验区建设。落实扶贫改革试验区建设总体方案。继续实行“一村一策”、“一户一法”，科学确定120条重点帮扶村帮扶项目；认真做好省直部门和兄弟市负责的164条重点帮扶村的协调服务和考核验收工作。继续推进互助金试点，积极探索保险扶贫，采取政府贴息的方式，支持困难群众融资发展生产。继续开展好“6·30广东扶贫济困日”活动，并详细公开捐款的去向和实际效果。

六、积极对接广州的全面帮扶与合作

建立对接帮扶与合作机制。制订一体化发展方案和对接帮扶工作方案。组建对口帮扶指挥部，成立重大事项专责小组，建立各县（市、区）与广州对应区（市）的对接帮扶机制，确定对接帮扶的具体内容，出台对接帮扶工作监督考核办法。按照大广州副中心城市的定位，编制交通、城市、产业布局、生态建设、公共服务等广清一体化发展规划。协助设立广州帮扶发展基金，启动广清区域性结算、融资平台建设。全面启动两市商业银行间跨行通存通兑及取消异地存、取款手续费工作。出台产业、财税、人才和社会发展对接帮扶政策。促进广清文化的交流与融合。

积极开展广清产业合作。完成对口帮扶产业项目目录，加快省级产业转移园与广州的对接，组织好广清产业对接3场园区推介活动。将广州（清远）产业园打造成广州开发区的清远园区，尽快完成园区运作架构、投资开发公司的组建，加快园区主干道路及配套设施建设，完成启动区交地工作，力争全年引进项目20个以上。完成源潭物流园总体规划和前期工作，建设白云国际机场空港物流服务中转基地，着力招引广州大型仓储物流企业进驻。推进“华南声谷”的规划建设和招商，打造广州现代服务业流程外包基地。完成“三连一阳”旅游合作区发展规划。推动广州农商行和广州银行落户清远。

充分对接广州的全面帮扶。启动英德、连州、佛冈等3个广州“市长菜篮子工程”建设。全面启动两市医疗保险参保人员异地就医费用即时结算和两地住房公积金互贷的协调对接。开展20所义务教育、30所学前教育学校的结对帮扶工作。通过广州医疗机构一对一辅导英德、连州市人民医院创建“三甲”﹝25﹞，并争取托管连山、连南、阳山县人民医院，帮助英德、佛冈、连南的5个镇中心卫生院提升医疗水平。抓好广州市对口帮扶连州市25个重点帮扶村、3244户贫困户的对接工作。

七、投入更多财力保障基本民生

加大医疗卫生事业投入。推进市级对县级医疗机构的技术扶持和托管工作。如期完成基本医疗卫生服务均等化年度任务。启动英德、连州和连山公立医院取消药品加成的综合改革。完成市中医院门（急）诊住院大楼主体建设，开工建设市人民医院二期工程，按“三甲”标准推动清新区人民医院建设，按“二甲”标准新建清城区人民医院。完成市职业病防治院和市精神病医院机构组建工作，启动慢性病职业病防治大楼和市精神病医院新院建设的前期工作。上半年落实各县（市、区）中心卫生院提升工程实施方案，每个县（市、区）启动1间中心卫生院升级改造工程。参照洲心创建国家示范社区卫生服务中心的经验，提升横荷、英城社区卫生服务中心及太和镇卫生院、北湖医院的服务能力。加强重点传染病特别是H7N9流感疫情的防控工作。

多管齐下提升教育水平。确保连州市创建成省教育强市，全市创建省教育强镇13个，实现省教育强县（市、区）、强镇（街、乡）的全覆盖。启动教育现代化先进县（市、区）创建工作。实现全市1150所公办学校及幼儿园“校校通”全覆盖；完成12所村小、94个教学点、30所农村寄宿制学校改造。实现免费义务教育公用经费补助标准小学每生950元/年，初中每生1550元/年。完善家庭经济困难学生资助体系。着力解决教师结构性缺编问题，确保山区和边远地区农村义务教育学校教师岗位津贴人均每月增加到700元。加强联片教研活动，建设100个校长、教师、班主任工作室。启动新一轮“学前教育三年行动计划”和特殊教育提升计划。启动省职教基地二期工程，确保岭南职院、碧桂园职院首期秋季落成开学。

加快推进文化体育设施建设。制定并实施文化体育基本公共服务均等化实施方案。改善基层文化体育基础设施，改扩建16个街镇文化站，完成278个行政村电子阅览室的建设。在有条件的乡村和社区规划建设体育公园，配建一批露天篮球场、乒乓球场等普惠型体育设施。推进基层文化场馆免费开放项目品牌化，完成市数字图书馆和省数字图书馆的无障碍连接。加强非物质文化遗产和文物保护，挖掘弘扬清远民间传统文化。出台繁荣文学文艺创作的实施方案。力争上半年完成市图书馆、篮球综艺馆、艺术中心、城市馆、科技馆、青少年宫等市级文化综合体的设计，下半年开工建设。同步开展文化场馆管理人才的招聘。

扎实做好劳动就业和社会保障工作。健全人才公共服务网络平台，深入实施校园对接产业园工程，力争新增城镇就业5万人，新增转移农村劳动力就业4万人。力争职工和城乡居民养老保险参保率分别达83%和99%，缴费率分别达81%和90%，基本医保参保率稳定在98%以上。落实城镇职工补充医疗保险政策，加快实现社保卡“一卡通”，力争社会保障卡户籍人口持卡人数达到365万人。加强退休人员社会化管理服务，企业退休人员社区管理服务率达到82%。适时启动临时价格补贴机制。

创新社会治理。加快推进“两建”信息综合管理平台建设。推广洲心“朝阳坊”、凤城居家养老等经验。健全公共安全体系，深入排查生产安全和食品药品安全隐患。开展地质灾害详查试点工作。认真实施全市一体的纠纷预警处置机制和企业失信曝光、工资保证金、欠薪应急周转金制度，实现劳动保障监察“两网化”管理覆盖率90%。坚持计划生育基本国策，提高出生人口素质，促进人口长期均衡发展。狠抓“平安清远”创建活动，加强社会治安综合治理，严厉打击各类违法犯罪活动，继续开展“打假”专项行动，推动公共责任保险，提高保险社会管理功能。建立常态化国防动员需求对接机制，做好国防教育、民兵预备役工作。继续做好双拥优抚安置工作，扎实推进全国双拥模范城创建工作。进一步抓好妇女儿童、民族宗教、外事侨务、残疾人、对台、审计、统计、气象、地震、档案、方志等工作。

办好十件民生实事。市、县两级分别投入3.19亿元和9.25亿元，集中力量办好十件民生实事。一是基本完成18条总里程260公里的国省道大修改造﹝26﹞；二是提高底线民生保障水平﹝27﹞，提高低保、五保、孤儿供养、医疗救助、残疾人补贴、基础养老金发放标准；三是完成保障性住房、棚户区改造住房2889套（户），新增发放廉租住房租赁补贴360户，完成6590户农村低收入住房困难户的住房改造；四是实施乡镇卫生院“五个一”设备装备提升工程；五是推进10宗中小河流治理项目建设;六是建设好全市85个镇（街、乡）网上办事分厅；七是在300个农村小学及教学点实施优质教学资源“班班通”工程；八是在市县镇三级改造50个以上肉菜市场；九是城乡建成59个社区体育公园；十是在市中心区域和部分县城增加13个公共停车场﹝28﹞。

八、加大生态保护和建设力度

加强北江流域保护治理。上半年制订出台北江流域水质保护和治理实施方案。年内完成北江流域饮用水源保护区养殖场、北江河道固定餐饮船的清理整顿，取缔库湾及河道上的瓷砂、洗矿厂；制订北江游船标准并对游船进行改造，规范餐厨垃圾管理；对北江沿线的城镇和产业园区的污水进行截污处理，提高北江流域的项目准入和产业门槛；加快白庙船厂搬迁；完成洲心、东城污水处理厂等国家污染减排责任书项目建设任务，抓紧推进污水处理厂配套管网和澜水河、龙塘河等截污工程及笔架河整治工程建设，确保北江水质不下降。

构建连阳生态发展试验区。抓紧出台主体功能区的人口、投资、绩效考核政策。通过加大生态发展考核权重，构建北部地区连绵山体森林生态屏障体系。在连阳地区和英德西部生态调节区探索生态发展新模式，争取成为国家生态文明建设示范点和国家级主体功能区规划实施示范市，并与湖南永州、广西贺州联合申报国家级南岭生态发展试验区。争取省财政加大对连阳地区和英西生态调节区的一般性转移支付力度。积极推动国家、省级项目免除民族自治地区配套资金。

实施新一轮绿化清远大行动。继续抓好生态景观林、森林碳汇林、森林进城围城等重点林业生态工程。建成森林公园37个、湿地公园5个、乡村绿化美化工程199个。大力培育推广乡土树种，打造省林业生态强市。结合建设森林公园，稳妥推进国有林场分类改革，解决林场职工收入来源问题。控制并逐步取缔市区规划范围内的矿山开采，扎实抓好关停矿山复绿。

加大节能减排工作力度。推进“省万家”、“市百家”企业节能低碳行动，完成75家重点耗能企业的节能考核工作，淘汰落后电机。建成市级能源管理中心平台，推进企业能耗在线监测。加快实施金属拆解业及陶瓷、水泥等企业的整治计划，逐家企业明确标准，规定达标时间。加强重金属污染减排工作。限期淘汰“黄标车”。加快推进主要污染物总量减排工作，确保完成国家减排责任书项目。强化依法用地、集约节约用地观念，加大土地管理执法力度，大力清理“三旧”改造项目，通过盘活存量用地来解决用地矛盾，提高土地利用水平。

各位代表，能否完成今年的各项任务，关键在于政府的执行力。我们将以开展党的群众路线教育实践活动为契机，进一步改进工作作风，真正做到为民、务实、清廉；我们将通过市、县两级机构的改革，更好地转变职能，提高效能；我们将提高依法行政水平，拓宽社会各界参与政府决策的渠道，使政府的公共决策和公共政策更符合公众的利益。我们将进一步加强廉政建设，坚持从源头上预防和治理腐败，重点加强对有行政许可权、执法权和公共资源支配权的部门和岗位的监管，以更大的力度整治潜规则，切实纠正部门和行业不正之风，防控廉政风险和执法风险。我们还将全力打造学习型政府，不断提升政府团队的综合素质和服务能力。

各位代表，我们深知，全市人民对清远的发展有更多的期待，每一位清远人都希望过上更好的生活。全市人民对幸福生活的向往和追求，就是我们工作的动力和努力的方向。我们只有竭尽全力，只争朝夕，才不会辜负全市人民的信任和期望。让我们在市委的坚强领导下，以更加务实实干的精神，大胆开拓进取，为创造清远更加美好的明天而努力奋斗！
